# ^11^C-UCB-J synaptic PET and multimodal imaging in dementia with Lewy bodies

**DOI:** 10.1186/s41824-020-00093-9

**Published:** 2020-12-22

**Authors:** Nicolas Nicastro, Negin Holland, George Savulich, Stephen F. Carter, Elijah Mak, Young T. Hong, Selena Milicevic Sephton, Tim D. Fryer, Franklin I. Aigbirhio, James B. Rowe, John T. O’Brien

**Affiliations:** 1 Department of Psychiatry, University of Cambridge, Cambridge, UK; 2 Division of Neurology, Department of Clinical Neurosciences, Geneva University Hospitals, 4 rue G. Perret-Gentil, 1205 Geneva, Switzerland; 3 Department of Clinical Neurosciences, University of Cambridge, Cambridge, UK; 4 Wolfson Brain Imaging Centre, University of Cambridge, Cambridge, UK

**Keywords:** Synaptic imaging, Dementia, Lewy bodies, Amyloid, Tau, Brain atrophy

## Abstract

**Objective:**

Dementia with Lewy bodies (DLB) is a common cause of dementia, but atrophy is mild compared to Alzheimer’s disease. We propose that DLB is associated instead with severe synaptic loss, and we test this hypothesis in vivo using positron emission tomography (PET) imaging of ^11^C-UCB-J, a ligand for presynaptic vesicle protein 2A (SV2A), a vesicle membrane protein ubiquitously expressed in synapses.

**Methods:**

We performed ^11^C-UCB-J PET in two DLB patients (an amyloid-negative male and an amyloid-positive female in their 70s) and 10 similarly aged healthy controls. The DLB subjects also underwent PET imaging of amyloid (^11^C-PiB) and tau (^18^F-AV-1451). ^11^C-UCB-J binding was quantified using non-displaceable binding potential (BP_ND_) determined from dynamic imaging. Changes in ^11^C-UCB-J binding were correlated with MRI regional brain volume, ^11^C-PiB uptake and ^18^F-AV-1451 binding.

**Results:**

Compared to controls, both patients had decreased ^11^C-UCB-J binding, especially in parietal and occipital regions (FDR-corrected *p* < 0.05). There were no significant correlations across regions between ^11^C-UCB-J binding and grey matter, tau (^18^F-AV1451) or amyloid (^11^C-PiB) in either patient.

**Conclusions:**

Quantitative imaging of in vivo synaptic density in DLB is a promising approach to understanding the mechanisms of DLB, over and above changes in grey matter volume and concurrent amyloid/tau deposition.

**Supplementary Information:**

The online version contains supplementary material available at 10.1186/s41824-020-00093-9.

## Introduction

Dementia with Lewy bodies (DLB) is a major cause of neurodegenerative dementia. It is characterized by recurrent visual hallucinations, parkinsonism, cognitive fluctuations and REM sleep behaviour disorder (McKeith et al., 2017). In contrast to clinical impairments, atrophy is typically mild in DLB compared to Alzheimer’s disease (AD). Instead, the severity of disease may arise from a severe loss of synapses. Autopsy findings in DLB include alpha-synuclein deposition in association with loss of synaptic proteins (Bereczki et al., 2016; Bajic et al., 2012). Synaptic density can be estimated in vivo from the synaptic vesicle protein 2A (SV2A, a vesicle membrane protein ubiquitously expressed in synapses) using the radioligand ^11^C-UCB-J with positron emission tomography (PET) (Nabulsi et al., 2016). In other degenerative disorders including AD, progressive supranuclear palsy and corticobasal degeneration, widespread reduction in ^11^C-UCB-J binding is observed, more so in disease-specific regions and in relation to clinical severity (Chen et al., 2018; Mecca et al., 2020; Holland et al., 2020).

In this case study, ^11^C-UCB-J PET imaging was assessed in two patients with probable DLB, with and without concurrent amyloid and tau deposition. We tested for differences in ^11^C-UCB-J binding between DLB and controls and assessed regional correlation of ^11^C-UCB-J binding with atrophy, PET imaging of amyloid (^11^C-Pittsburgh compound B (PiB)) and tau (^18^F-AV1451).

## Material and methods

### Patients inclusion

We included two patients with probable DLB: (1) a male presenting with a 5-year history of cognitive impairment (revised Addenbrooke’s Cognitive Evaluation (ACER) score 81/100) interfering with daily activities, in association with cognitive fluctuations, REM sleep behaviour disorder and parkinsonism (Movement Disorders Society – Unified Parkinson’s Disease Rating Scale (MDS-UPDRS) part III 27/132) and (2) a female presenting with a 2-year cognitive impairment (ACER 50/100) associated with cognitive fluctuations, visual hallucinations and parkinsonism (MDS-UPDRS III 13/132). Patients were recruited from specialist memory clinics in and around Cambridgeshire, the Dementias and Neurodegeneration specialty of the UK Clinical Research Network (DeNDRoN) or the Join Dementia Research (JDR) platform (www.joindementiaresearch.nihr.ac.uk). Probable DLB was defined by the 2017 consensus criteria (McKeith et al., 2017).

Both patients underwent PET imaging with ^11^C-UCB-J, ^11^C-PiB and ^18^F-AV1451, as well as structural magnetic resonance imaging (MRI) (Bevan-Jones et al., 2017). They were compared to ten similarly aged control subjects, who were recruited from the JDR and local registers. Healthy controls had MMSE > 26, no cognitive symptoms, unstable/significant medical history or MRI contraindications.

### PET and MRI imaging acquisition and preprocessing

All radioligands were prepared at the Wolfson Brain Imaging Centre, University of Cambridge (Milicevic-Sephton et al., 2020). ^11^C-UCB-J PET imaging (mean injected activity: 368 MBq controls; 325 MBq DLB cases) used dynamic scanning for 90 min on a GE SIGNA PET/MR (GE Healthcare, Waukesha, USA), with attenuation correction including the use of a multi-subject atlas method (Burgos et al., 2014; Wu & Carson, 2002). For the DLB patients only, additional static ^11^C-PiB (550 MBq) and dynamic ^18^F-AV-1451 (370 MBq) PET scans were performed. Each emission image series was aligned using SPM12 (https://www.fil.ion.ucl.ac.uk/spm/software/spm12) to ameliorate the impact of patient motion during data acquisition and then rigidly registered to the corresponding T1-weighted MRI image. The Hammersmith atlas (http://brain-development.org/brain-atlases) regions of interest (ROIs) were non-rigidly registered to each T1-weighted MRI image. Regional time-activity curves were corrected for cerebrospinal fluid partial volume using SPM12 tissue probability maps smoothed to PET spatial resolution. ^11^C-UCB-J non-displaceable binding potential (BP_ND_) was determined using a basis function implementation of the simplified reference tissue model (Wu & Carson, 2002), with the reference tissue defined in the centrum semiovale (Koole et al., 2019). Regional ^11^C-PiB standardized uptake value ratio (SUVR) was determined using cerebellar grey matter as the reference tissue, which was also used as the reference region for ^18^F-AV-1451 BP_ND_ quantified using a basis function simplified reference tissue model.

MRI imaging used a 3T scanner (MAGNETOM Trio; Siemens Healthineers, Erlangen, Germany) including a magnetization-prepared rapid gradient echo (MPRAGE) T1-weighted sequence (repetition time = 2300 ms, echo time = 2.98 ms, field of view = 240 × 256 mm^2^, 176 slices, flip angle = 9°, isotropic 1 mm voxels). Grey matter volume was assessed by volume-based morphometry (VBM) with Computational Anatomy Toolbox 12 (CAT12) using the standard pipeline (http://www.neuro.uni-jena.de/cat) (Dahnke et al., 2013). Images were smoothed with the recommended 8 mm-full-width at half maximum Gaussian kernel and regional values were extracted from the Hammersmith atlas.

### Statistical analysis

ROI-based comparison of ^11^C-UCB-J BP_ND_ and VBM data between the two DLB subjects (1/2 (50%) male, mean age 73 years) and 10 similarly aged controls (5/10 (50%) male, age mean ± SD 72.4 ± 3.4 years) was performed using a general linear model with repeated measure ANCOVA using age, sex and years of education as covariates. As 33 cortical and subcortical ROIs were assessed, we used a false discovery rate (FDR)-corrected *p* < 0.05 with *Q* = 0.15. Subsequently, regional *Z*-scores for ^11^C-UCB-J BP_ND_, ^11^C-PiB SUVR, ^18^F-AV1451 BP_ND_ and VBM data were computed for the DLB subjects by comparing them to the control group. Region-by-region correlations between the different brain imaging modalities were performed for the DLB subjects using Spearman correlations.

## Results

**Fig. 1:**
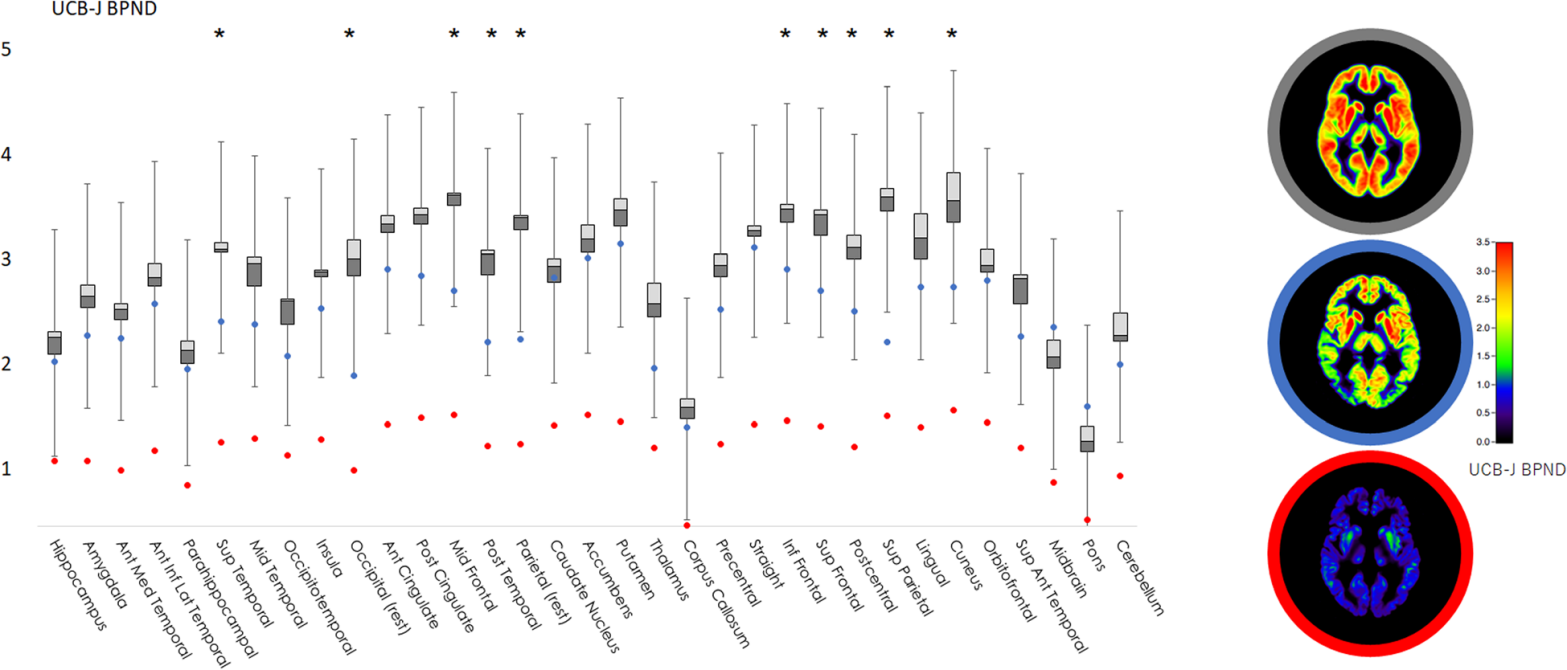
Box-and-whisker plot showing the regional ^11^C-UCB-J binding for the control group (*grey*) and scatter plot for DLB patient 1 (amyloid-negative, *red*) and DLB patient 2 (amyloid-positive, *blue*). Asterisk symbol indicates the regions with significant decrease for DLB (FDR-corrected *p* < 0.05). On the right, mean axial ^11^C-UCB-J scan for controls (*grey medallion*), DLB patient 1 (*red*) and DLB patient 2 (*blue*)

The first DLB patient (74-year-old male) was amyloid-negative (average neocortical ^11^C-PiB SUVR = 1.3), while the second patient (72-year-old female) was amyloid-positive (SUVR = 1.9). For ^11^C-UCB-J, there were significant group (*p* = 0.0012), region (*p* < 0.0001) and group × region interaction effects (*p* < 0.0001). Pairwise regional comparisons revealed decreased ^11^C-UCB-J BP_ND_ for both DLB patients compared to controls in extensive cortical regions (10/33 regions) and thalamus, with more prominent changes in parieto-occipital, middle and superior frontal cortices (FDR-corrected *p* < 0.05) (Fig. 1). Similar results were obtained using non-partial-volume-effect corrected data. ^11^C-UCB-J time-activity curves in the reference region (centrum semiovale) were similar among groups (Supp. Figure 1). Of note, we did not observe any significant differences between DLB and controls in the deep brain nuclei, except for the thalamus (FDR-corrected *p* < 0.05).

VBM analysis of regional volumes showed a significant effect of region (*p* < 0.0001) but no significant effect of group (*p* = 0.17) or group × region interaction (*p* = 0.20). In addition, we observed that ^11^C-UCB-J BP_ND_ *Z*-scores for DLB subjects were lower compared to VBM *Z*-scores. For example, in the superior frontal gyrus, ^11^C-UCB-J *Z*-score was − 1.7 while grey matter atrophy *Z*-score was − 0.4. Similarly, ^11^C-UCB-J BP_ND_ *Z*-score in lingual gyrus was − 1.5 whereas VBM *Z*-score was − 0.7.

Region-by-region correlations for the DLB patients showed no significant correlation between ^11^C-UCB-J BP_ND_ and grey matter volume (rho = 0.24, *p* = 0.17 for DLB patient 1 and rho = 0.27, *p* = 0.13 for DLB patient 2). No significant correlation was observed between ^11^C-PiB SUVR and UCB-J BP_ND_ (both patients *p* > 0.3). ^18^F-AV1451 BP_ND_ and ^11^C-UCB-J BP_ND_ did not correlate in either patient (*p* > 0.10).

## Discussion

The present study indicates marked synaptic loss in DLB using ^11^C-UCB-J PET. Reduced ^11^C-UCB-J binding was evident in posterior cortical regions after partial volume effect correction and so represented a change beyond that of brain atrophy. This demonstrates markedly reduced SV2a density in DLB. Further studies are needed at earlier prodromal stage to understand the progression of synaptic loss in DLB.

Previous findings in MCI and early AD indicated synaptic loss restricted to the hippocampus (Chen et al., 2018), while in AD decreases in ^11^C-UCB-J binding were found in the hippocampus, amygdala, thalamus, cingulate and temporal lobe (Venkataraman, 2019). Recently, widespread synaptic loss was observed in cortical and subcortical regions for 4-R tauopathies (Holland et al., 2020). Toyonaga et al. showed that in an amyloid precursor protein and presenilin 1 double transgenic mice model of AD, ^11^C-UCB-J baseline PET exhibited decreased hippocampal synaptic density, while the follow-up scan after treatment with saracatinib (a dual kinase inhibitor) revealed a significant increase in hippocampal synaptic density (Toyonaga et al., 2019). These findings suggest that ^11^C-UCB-J could be a valuable marker of disease severity and treatment monitoring for future therapeutic trials in human.

Regarding Parkinson’s disease (PD), Matuskey et al. recently showed that a significant (17–41%) reduction of ^11^C-UCB-J binding was observed for mild-moderate PD in deep nuclei (substantia nigra, red nucleus, locus coeruleus) and parahippocampal gyrus (Matuskey et al., 2020). For the present study, our DLB did not show any significant changes in synaptic density in deep brain nuclei, except for the thalamus. However, it is worth mentioning that we performed correction for multiple comparisons, which was not the case for the study by Matuskey et al.

In our two cases, we did not observe correlations between regional ^11^C-UCB-J BP_ND_ and regional atrophy, suggesting that our assay of synaptic loss is not merely an index of volume loss. Regional synaptic density loss was also not associated with amyloid (^11^C-PiB) or tau (^18^F-AV1451) in both DLB patients, suggesting that the synaptic ligand is informative over synaptic aspects of pathogenesis over and above the information obtained from amyloid and tau imaging.

Our study has limitations, including the inclusion of a case and replication rather than a large cohort. In addition, the synaptic PET was obtained after the tau and amyloid PET, and we cannot exclude progression of pathology even in the absence of a significant progression of the clinical severity. Moreover, arterial input function was not performed during the acquisition of PET imaging. We also rely on clinical diagnostic criteria and do not have pathological confirmation of DLB pathology.

## Conclusions

In summary, we report that the binding of ^11^C-UCB-J—an in vivo marker of synaptic density—was markedly decreased in two well-characterized DLB patients. Further studies are required to assess whether such changes are characteristic of DLB, to correlate synaptic loss with key symptoms and to monitor change over time from prodromal through to established dementia in order to improve our understanding of pathophysiological processes involved in neurodegeneration.

## Supplementary Information

**Additional file 1:** **Supplementary Figure 1.** ^11^C-UCB-J time-activity curves in the reference region.

## Data Availability

The datasets used and analysed during the current study are available from the corresponding author on reasonable request.

## Abbreviations

ACER: Addenbrooke’s Cognitive Evaluation Revised
AD: Alzheimer’s disease
BPND: Non-displaceable binding potential
CAT12: Computational Anatomy Toolbox 12
DLB: Dementia with Lewy bodies
FDR: False discovery rate
MDS-UPDRS: Movement Disorders Society Unified Parkinson Disease Rating Scale
MRI: Magnetic resonance imaging
PiB: Pittsburgh compound B
PET: Positron emission tomography
SUVR: Standardized uptake value ratio
SV2A: Synaptic vesicle 2A
