# Supplementary figures and images for: 11C-UCB-J synaptic PET and multimodal imaging in dementia with Lewy bodies
Source: Eur J Hybrid Imaging. 2020 Dec 22;4:25. doi: 10.1186/s41824-020-00093-9 (PMC7752786; doi:10.1186/s41824-020-00093-9)

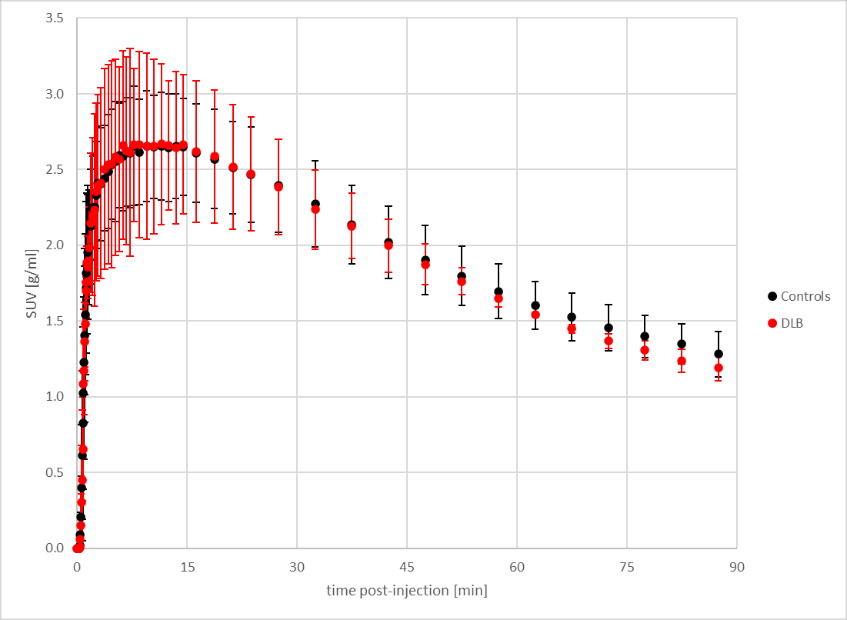

Supplement: Supplementary file 1 — Additional file 1: Supplementary Figure 1. 11C-UCB-J time-activity curves in the reference region. [file 41824_2020_93_MOESM1_ESM.png]
